# Alpha band modulation caused by selective attention to music enables EEG classification

**DOI:** 10.1007/s11571-023-09955-x

**Published:** 2023-04-07

**Authors:** Kana Mizokuchi, Toshihisa Tanaka, Takashi G. Sato, Yoshifumi Shiraki

**Affiliations:** 1 Department of Electrical and Electronic Engineering, Tokyo University of Agriculture and Technology, Tokyo, Japan; 2 Department of Electrical Engineering and Computer Science, Tokyo University of Agriculture and Technology, Tokyo, Japan; 3 NTT Communication Science Laboratories, Nippon Telegraph and Telephone Corporation, Kanagawa, Japan

**Keywords:** Selective attention, Electroencephalogram (EEG), Music, Alpha band, Entrainment

## Abstract

Humans are able to pay selective attention to music or speech in the presence of multiple sounds. It has been reported that in the speech domain, selective attention enhances the cross-correlation between the envelope of speech and electroencephalogram (EEG) while also affecting the spatial modulation of the alpha band. However, when multiple music pieces are performed at the same time, it is unclear how selective attention affects neural entrainment and spatial modulation. In this paper, we hypothesized that the entrainment to the attended music differs from that to the unattended music and that spatial modulation in the alpha band occurs in conjunction with attention. We conducted experiments in which we presented musical excerpts to 15 participants, each listening to two excerpts simultaneously but paying attention to one of the two. The results showed that the cross-correlation function between the EEG signal and the envelope of the unattended melody had a more prominent peak than that of the attended melody, contrary to the findings for speech. In addition, the spatial modulation in the alpha band was found with a data-driven approach called the common spatial pattern method. Classification of the EEG signal with a support vector machine identified attended melodies and achieved an accuracy of 100% for 11 of the 15 participants. These results suggest that selective attention to music suppresses entrainment to the melody and that spatial modulation of the alpha band occurs in conjunction with attention. To the best of our knowledge, this is the first report to detect attended music consisting of several types of music notes only with EEG.

## Introduction

In our daily lives, we are constantly exposed to music. We naturally and selectively attend to specific music sources while ignoring others, even when multiple sources are playing simultaneously. To do this, humans have the ability to selectively attend to a certain auditory source (Pugh et al. 1996; Karns et al. 2015). It is crucial to understand the neural mechanisms of attention to specific music and to decode which music we are attending to by physiological measurements in engineering applications.

The response of the brain to auditory stimuli such as speech and music can be measured by electroencephalography (EEG) (Hill et al. 2005; Horton et al. 2013; Meltzer et al. 2015; Dong et al. 2017; Stupacher et al. 2017; Kumagai et al. 2018; Baltzell et al. 2019), electrocorticography (Sturm et al. 2014), functional magnetic resonance imaging (Trost et al. 2014), magnetoencephalography (Doelling and Poeppel 2015), and other techniques. Of these, EEG is a noninvasive measurement method with a high temporal resolution (Dietrich and Kanso 2010), which makes it appropriate for analyzing responses to auditory stimuli, including high-frequency components. One crucial response is neural entrainment, which refers to low-frequency cortical neural oscillations coupled with acoustic dynamics when speech or music are perceived (Lakatos et al. 2013; Doelling and Poeppel 2015; Meltzer et al. 2015; Stupacher et al. 2017; Kumagai et al. 2018; Baltzell et al. 2019; Wollman et al. 2020). Specifically, the envelope of auditory stimuli tends to modulate the cortical activity to become coupled with low-frequency oscillations.

These types of brain responses are elicited by selective attention when a human perceives several auditory modalities, such as speech and music (Choi et al. 2013; Horton et al. 2013; Kong et al. 2014; Meltzer et al. 2015). There have been many reports on the mechanisms of selective attention with respect to speech (Kerlin et al. 2010; Horton et al. 2013, 2014; Meltzer et al. 2015). Horton et al. (2013) found that when participants listened to two speeches, the cross-correlation seen from the EEG signal with the attended speech was stronger than that with the unattended speech. For music, Meltzer et al. (2015) conducted an experiment in which the participants listened to music composed of only quarter notes of the same length. Their results showed that the neural entrainment between the beat of the melody and the EEG signal was stronger when the participants attended to the stimuli than when they did not.

Another important factor in comprehending attention is the spatial modulation of brain activity (Alho et al. 1999; Kerlin et al. 2010; Liu et al. 2016; Dong et al. 2017; Kumagai et al. 2018; Cai et al. 2021). Kerlin et al. (2010) measured EEG signals during selective attention to two speeches. They showed that change in attention was indicated as a difference in alpha power at the parietal areas across hemispheres. Moreover, Kumagai et al. (2018) and Matsui and Tanaka (2019) used the common spatial pattern (CSP) method (Ramoser et al. 2000) to identify the attended object from EEG signals. These studies succeeded in distinguishing the condition between visual and auditory attention and the condition between musical and speech attention, suggesting that attention influences spatial modulation. However, the effect of selective attention on the entrainment and spatial modulation in EEG signals when listening to multiple pieces of music is unclear.

Based on the above findings, we hypothesized the following effects of selective attention to music. The first hypothesis is that the cross-correlation of the EEG signal with attended music is different from that with unattended music. The second hypothesis is that the spatial modulation of the EEG response is different depending on the attentional condition. To investigate these two hypotheses, we measured the EEG signals when a human paid selective attention to one of two melodies. We used the cross-correlation function between the EEG signals and envelopes of stimuli to evaluate neural entrainment. Moreover, the spatial modulation of the response was evaluated based on a machine learning approach whereby the CSP method was applied.

## Materials and methods

### Participants

Fifteen healthy young adults (mean age 21.73 years; range 20–23 years; 7 females) participated. One of the participants was left-handed. All participants were self-reported that they had normal hearing and no musical training. Some of the participants were lab students. Non-lab participants received a payment of JPY 1,000 per hour. None of the student participants were encouraged to participate in this experiment by their professors, nor did they obtain any credits for doing so. They gave their written informed consent. This study was approved by the institution’s Research Ethics Committee (approval no. 31–51, 31–59).

**Table 1:** Two combination patterns of auditory stimuli

	Left channel	Right channel
Instrument	Harpsichord	Piano
Tempo	127 bpm (about 2.1 Hz)	191 bpm (about 3.2 Hz)
Pattern 1	“Twinkle Twinkle Little Star” Note length: mean 0.540 s, s.d. 0.165 s	“Ode To Joy” Note length: mean 0.324 s, s.d. 0.101 s
Pattern 2	“Go Tell Aunt Rhody” Note length: mean 0.319 s, s.d. 0.173 s	“Csikos Post” Note length: mean 0.178 s, s.d. 0.100 s

**Fig. 1:**
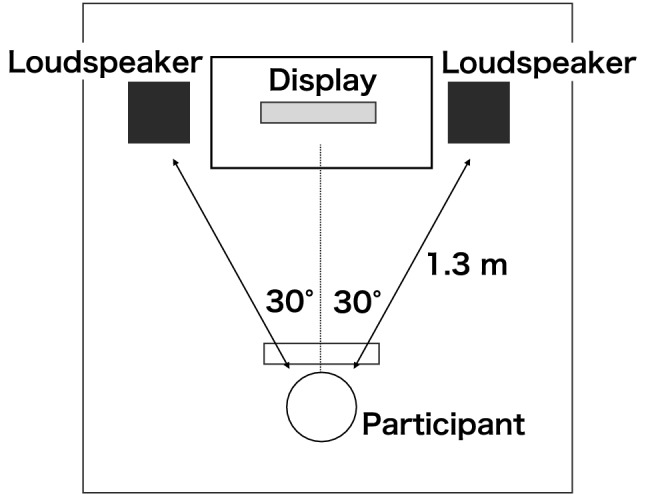
Experimental environment

### Stimuli

To investigate the effect of selective attention to music, we used auditory stimuli consisting of a beep for 100 ms with a pitch of 440 Hz followed by two different musical excerpts. Each excerpt was presented from the left and right loudspeakers or earphones. The beep and the stimulus music were recorded in the same sound file (.wav). The beep signal was used to determine the onset time of the stimulus and EEG recording.^1^

We selected the following four well-known pieces of music: “Twinkle Twinkle Little Star,” “Go Tell Aunt Rhody,” “Song of Joy,” and “Csikos Post.” These pieces were in MIDI format and created with Sibelius (Avid Technology, USA) and Python to set an arbitrary and consistent tempo. We presented two different types of combination patterns of the musical pieces, as listed in Table 1, where the statistics of the length of musical notes are also given. Each pattern consisted of two musical excerpts with different instruments (harpsichord and piano) and different tempos (127 and 191 beats per minute, bpm). In the above setting, the half, quarter, eighth, and sixteenth notes amounted to 1.1, 2.1, 4.2, and 8.5 Hz (notes per second) for 127 bpm and 1.6, 3.2, 6.4, and 12.7 Hz for 191 bpm, respectively. All audio files were created with a sampling frequency of 44,100 Hz.

We put silent moments in the attended melody to ensure that the participants attended to the indicated excerpt. The duration of the silence was 500 ms (100 ms rise and fall time). The attended melodies had two to four silent intervals randomly per trial (60 s or 59 s). All musical excerpts except “Csikos Post” were less than 60 s and, therefore, were repeatedly presented for a duration of 60 s or 59 s.

The stimuli were presented in two different manners: a loudspeaker (705S2, B &W, UK) for the first day and an earphone (ER–4 S, Etymotic Research, USA) for the second day. The loudspeakers were placed at $\pm 30^\circ$, 1.3 m from the participants, as shown in Fig. 1 (Bregman 1994).

**Fig. 2:**
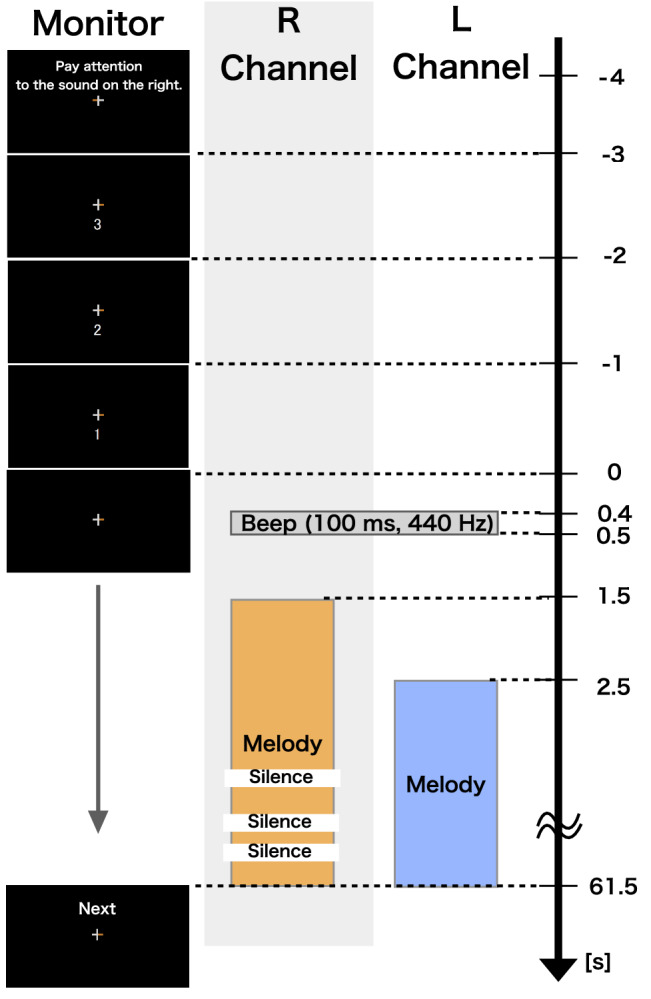
Experimental flow (in the case of the right-attended condition, in which the participants paid attention to the auditory stimuli from the right)

### Procedure

The experiment consisted of two conditions, “right-attended” and “left-attended,” in which the participants paid attention to the auditory stimuli from the right and the left, respectively. We presented music stimuli with the loudspeakers on the first day and with the earphones on the second day. The interval between the first and second experimental day was more than two weeks (Meltzer et al. 2015). As a behavioral experiment to check the participants’ concentration on the attended melody, they were instructed to press a button when the attended melody became silent.

At the beginning of the experiment, we asked the participants whether they were familiar with the musical excerpts. Each day, the participants were given practice time before the experiment to become familiarized with the task. During this practice time, the participants first listened to each musical excerpt before listening to the auditory stimulus and finally practiced pressing the buttons when they found a silent moment in the attended stream.

We conducted the experiment in a soundproof room (AMDB20H, Yamaha, Japan) with a sound insulation performance of $-35$ dB to prevent the influence of outside noise. The soundproof room was used to shield the participants from external noise. We instructed the participants not to move their bodies, except for their fingers to press the button during the task and their heads to fix their position. We also instructed them to hold the button for the behavioral experiment in their right hand.

An example of a single trial (the right-attended condition) is shown in Fig. 2. We used a monitor (VX279, ASUS, Taiwan) for displaying the instructions. First, the directions regarding attention (right or left) were given on the screen for a second. A fixation cross was presented at the center of the screen, and then a countdown in seconds to the beginning of the task was displayed at the bottom of the cross. The left or right part of the fixation cross was filled out in orange to highlight the instruction for attention, as illustrated in Fig. 2.

After the cue ($t=0$ s), the beep was presented for 0.1 s ($t=0.4$ – 0.5 s). The beep onset was used for defining the absolute time for the analysis. Then the attended melody was presented for 60 s, starting at $t=1.5$ s, and the unattended melody was presented for 59 s starting at $t=2.5$ s, as shown in Fig. 2. After $t=3.5$ s, the behavioral task began. Thus, each auditory stimulus lasted 61.5 s from the cue, including the beep.

One block consisted of seven trials. The two stimulus patterns were presented in an alternating fashion in each trial. Half the participants started from the right-attended condition, and the others started from the left.

The participants were given a five-minute rest after the end of each block. The block was repeated four times, implying that a total of 56 trials were conducted. The two conditions (right- and left-attended) were presented in an alternating fashion in each block.

### Data recording

EEG signals were measured using 64 scalp Ag/AgCl passive electrodes mounted in an EEG gel head cap (TMSi; Twente Medical Systems International, Oldenzaal, the Netherlands) and based on the international 10–10 system (Fp1, Fpz, Fp2, F7, F5, F1, F3, Fz, F2, F4, F6, F8, FC5, FC3, FC1, FCz, FC2, FC4, FC6, M1, T3, C5, C3, C1, Cz, C2, C4, C6, T4, M2, CP5, CP3, CP1, CPz, CP2, CP4, CP6, T5, T6, P5, P3, P1, Pz, P2, P4, P6, O1, Oz, O2, AF7, AF3, AF4, AF8, PO7, PO5, PO3, POz, PO4, PO6, PO8, FT7, FT8, TP7, and TP8). The impedance of all the electrodes was kept below 10 k$\Omega$.

In addition, signals around the eye were recorded simultaneously with the EEG signals with two electrodes placed on the top of the right eye (referenced to the left earlobe) and next to the outer canthus of the eye (referenced to the right earlobe) to detect blink and eye movement. The ground electrode was placed on the left wrist. All channels were amplified using a Refa 72-channel amplifier (TMSi) against the average of all connected inputs. The signals were sampled at a sampling rate of 2048 Hz and recorded using Polybench (TMSi). For synchronization of auditory stimuli with biological signals and the behavioral experiment, audio and button signals were recorded by the Refa amplifier through a Dual Channel Isolation Amplifier (TMSi).

### Data analysis

#### Preprocessing

First, the recorded EEG signal was re-referenced to the average potential of the M1 and M2 electrodes. Second, we applied an infinite impulse response notch digital filter (50 Hz) and a ninth-order Butterworth digital highpass filter (0.4 Hz). Third, to remove any artifacts caused by either eye movement or blinking, we applied a blind source separation algorithm, called second-order blind identification (Belouchrani et al. 1993), to the re-referenced 62-ch EEGs two-ch signals around the eye. We then rejected estimated source signals that were highly correlated to either two signals around the right eye.^2^ Moreover, the EEG signals filtered by a low-pass filter with a cutoff frequency of 100 Hz were downsampled to 256 Hz. Then a fifth-order Butterworth digital bandpass filter between 0.4 Hz and 40 Hz was applied (Lalor et al. 2022). It should be noted that EEG signals were excluded from the analysis for five seconds after the onset of the silent interval (500 ms) and five seconds after the button was pressed.

#### Frequency analysis

To see the frequency response of the EEG signals, we calculated the averaged spectral densities (periodogram with a boxcar function) of the EEG signals (3.5-$-$58.5 s) across trials. We also obtained the grand average, the spectral densities averaged across all electrodes and participants.

#### Correlation analysis

To study neural entrainment, we calculated the cross-correlation function between the envelope of the music and the EEG signal similarly as in the study by Kumagai et al. (2018).

We calculated the envelope as follows. The original music signals were first resampled for the auditory stimuli from 44,100 to 8192 Hz. After doing this, we calculated the envelopes of the resampled auditory stimuli using Hilbert transform. Then we filtered the envelopes using a low-pass filter with a cutoff frequency of 100 Hz and downsampled the envelopes to 256 Hz. Finally, a zero-phase fifth-order Butterworth digital bandpass filter between 0.4 and 40 Hz was applied to the envelope.

The cross-correlation function is expressed as follows:

1 $$\begin{aligned} \mathrm {Cross-correlation}(\textrm{ch}, \tau ) = \sum _{t}{\textrm{env}(t) \textrm{eeg}(\textrm{ch}, t + \tau )}, \end{aligned}$$

where $\textrm{env}(\tau )$ and $\textrm{eeg}(\textrm{ch}, \tau )$ denote the normalized melody envelope and the normalized EEG signal at a given time instance $(t)$ and channel $\textrm{ch}$, respectively. Also, $\tau$ denotes the lag between the melody envelope and the EEG signal. The lag $\tau$ was set from $-0.625$ to $0.625$ s. The time step of $\tau$ was 1/256 s since the sampling frequency was 256 Hz. The analyzed EEG signal was a duration of 58 s (from $t=3.5$ to 61.5 s). For 5 s after silence onset, the EEG values in the calculation of (1) were set to 0 to eliminate the effect of event-related responses to the appearance of the silence.

#### Statistical analysis

**Fig. 3:**
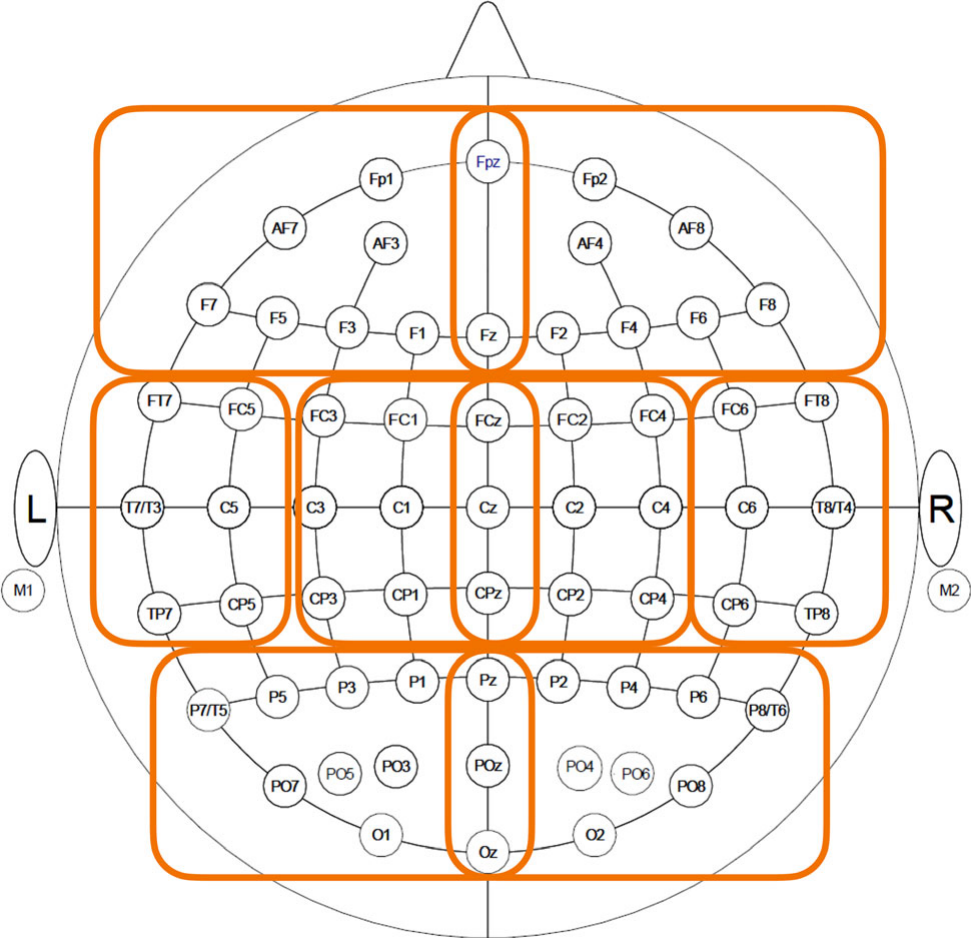
Grouping the electrodes to eight regions in the *t* test

For the cross-correlation function, we conducted three types of evaluation tests. First, we investigated the effect of the cross-correlation function between the EEG signals and music stimuli *not* presented simultaneously. This was done to examine if the amplitude of the cross-correlation function reflected entrainment between the rhythm of the auditory stimuli and EEG signals. Second, we examined the cross-correlation function across the attention conditions and presentation methods (i.e., loudspeaker and earphone). Finally, we investigated the cross-correlation function across the attention conditions for each region of the brain.

In the first analysis, as suggested by Zoefel and VanRullen (2016) and Kumagai et al. (2018), we compared the cross-correlation function between the EEG signals and the musical stimuli presented simultaneously with surrogate distributions; the surrogate distributions were given as cross-correlation functions between the EEG signal for each trial and the envelope of musical stimuli *not* presented. For example, in the case of EEG signals, while listening to the stimulus of Pattern 1, we used the stimulus of Pattern 2, which was not listened to, for the surrogate distributions. We generated 7500 envelope patterns by shuffling the melodies into each measure. We calculated the p-values for each sample point of the cross-correlation function using surrogate distributions averaged across electrodes. The null hypothesis is that the electrode mean of the real distribution is equal to the electrode mean of the surrogate distribution. The sample sizes of real distributions were the number of trials (i.e., about 800 trials by each attention condition type and presentation method of stimuli).

In the second analysis, we performed a two-way repeated-measures analysis of variance (ANOVA) tests on peak values of the cross-correlation function in attentional conditions and presentation methods. The independent variables were the attention condition and presentation methods, and the peak values averaged across the electrodes in the individual participants were the dependent variable. The sample sizes were the number of participants (i.e., loudspeaker, 14; earphone, 15).

In the third analysis, we applied a paired *t* test for each of the eight brain regions, as shown in Fig. 3. The regions corresponded to the left and right hemispheres and frontal, central, temporal, and parietal/occipital areas. The null hypothesis is that the average peak value in the attended condition is equal to that in the unattended condition. The *t* test used values averaged across presentation methods, electrodes, and trials for each brain region in the individual participants. In other words, the sample size for the t-test was the same as the number of participants.

#### Classification based on common spatial patterns

**Fig. 4:**
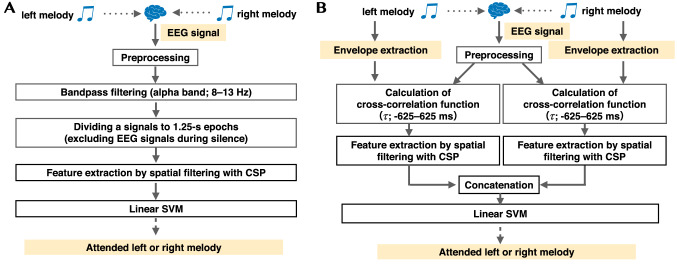
The block diagram of the machine learning classification experiment. (**A**) is using only the EEG signal, and (**B**) is using the cross-correlation function between the auditory stimulus and EEG signals

We conducted a machine learning classification experiment based on the hypothesis that differences in attention to different music manifest themselves in differences in the spatial distribution of alpha bands, as shown in Fig. 4. The spatial pattern used here is the common spatial pattern (CSP) (Ramoser et al. 2000), in which the variance of the weighted average EEG in the CSP is the largest between the two conditions.

Although the CSP method was initially developed to differentiate the spatial patterns of EEG signals during motor–imagery tasks (Ramoser et al. 2000), recent EEG studies have suggested that the CSP method also discriminates attention in the visual or auditory modality (Kumagai et al. 2018; Cai et al. 2021). The current paper examined two scenarios: estimating CSPs from a) only the EEG signal and b) the cross-correlation function between the auditory stimulus and EEG signals.

The CSP method calculates a filter with spatial weights corresponding to each electrode of the EEG signal (Ramoser et al. 2000). The spatial weights are the largest and smallest eigenvectors of the eigenvalue vectors obtained by solving the generalized eigenvalue problem of the covariance matrix obtained from each condition’s EEG signal or cross-correlation function. By projecting the EEG signal or cross-correlation function with a filter whose coefficients are the spatial weights, a signal is obtained that maximizes the variance between the two conditions. By calculating this signal’s temporal variance and computing the variance logarithm, we could obtain the feature for a classifier (Ramoser et al. 2000; Kumagai et al. 2018).

For the first scenario of using only EEG signals in the CSP method, we extracted the alpha band of 8–13 Hz and divided the EEG signals between 3.5 and 61.5 s into 1.25 s epochs (Ramoser et al. 2000). Each epoch was labeled “left” or “right” (attended). Then four log-variance features corresponding to the first two largest eigenvalues and the last two smallest eigenvalues were extracted from the EEG signal.

For the second scenario of using the cross-correlation function, we calculated a cross-correlation function between each segmented EEG signal epoch and the corresponding envelope of the right melody, as in (1). In the same way, we obtained a cross-correlation function with the envelope of the left melody. Each cross-correlation function was projected to a vector consisting of two log-variance features corresponding to the first largest eigenvalues and the last smallest eigenvalues by the CSP method. In other words, the feature vector for each EEG signal epoch consisted of four log-variance features.

We evaluated the classification accuracy through fivefold cross-validation using the linear SVM in both scenarios. Furthermore, in the same manner, we repeated the fivefold cross-validation 2000 times using randomly labeled data to ensure that the classification results were not because of chance (Kumagai et al. 2018).

## Results

### Behavioral analysis

**Fig. 5:**
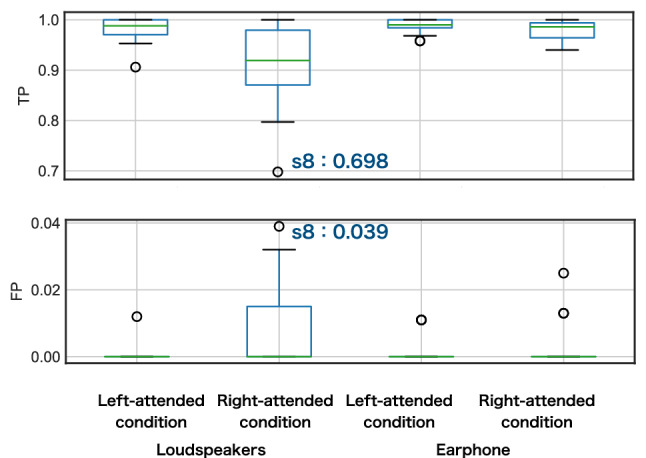
The result of the behavioral experiment. TP and FP stand for true positive and false positive, respectively. Participant s8 was excluded from the following analysis due to the low accuracy of its answers

According to the survey, all participants confirmed that they knew the musical excerpts. Figure 5 shows the ratio of button presses during silence (true positive, TP) and the ratio of button presses during nonsilence (false positive, FP), which represents if the participants performed the task correctly. The results showed that the percentage of correct answers was below 0.7 in the right condition for Participant s8 with loudspeakers, so this was excluded from the following analysis. Also, one trial with Pattern 2 in the right condition was excluded because of a technical error in creating the auditory stimuli for the presentation.

### Frequency analysis

**Fig. 6:**
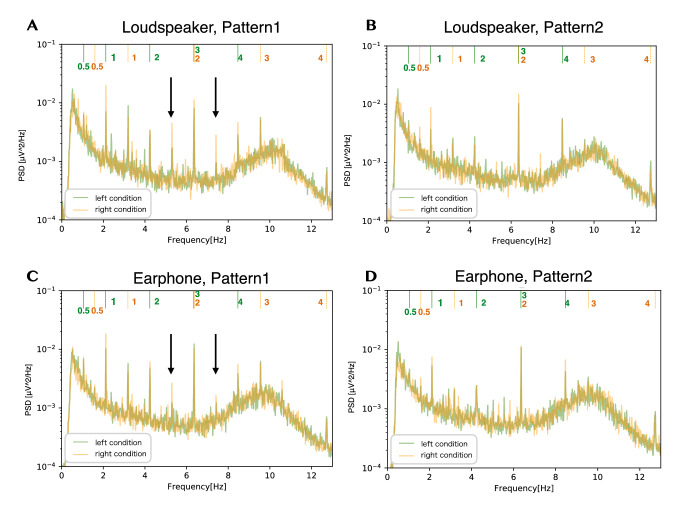
Spectral density averaged across trials, all electrodes, and participants in the experiment. The auxiliary lines are drawn vertically at frequencies that are the harmonics of the melody’s tempo. The auxiliary line for the melody presented from the left side is green, and the auxiliary line for the melody presented from the right side is orange. As indicated by solid arrows, we observed peaks at approximately 5.3 Hz and 7.4 Hz, which are not the harmonics of the tempo

We calculated the spectral density of EEG signals while listening to the musical stimulus. The spectral densities of EEG signals averaged across trials, all electrodes, and participants in the experiment are shown in Fig. 6. Auxiliary lines are drawn vertically under the top of each figure, and the numbers indicate the harmonics of each melody and its index. As shown in Fig. 6, we can observe the clear peaks at the frequencies of the harmonics of the melody. Also, the strongest peak tends to occur at 6.4 Hz, which was not the fundamental tempo in Pattern 2. In addition, in Pattern 1, we can observe peaks at approximately 5.3 Hz and 7.4 Hz (as indicated by solid arrows in Fig. 6), which were not the harmonics of the tempo.

### Correlation analysis

**Fig. 7:**
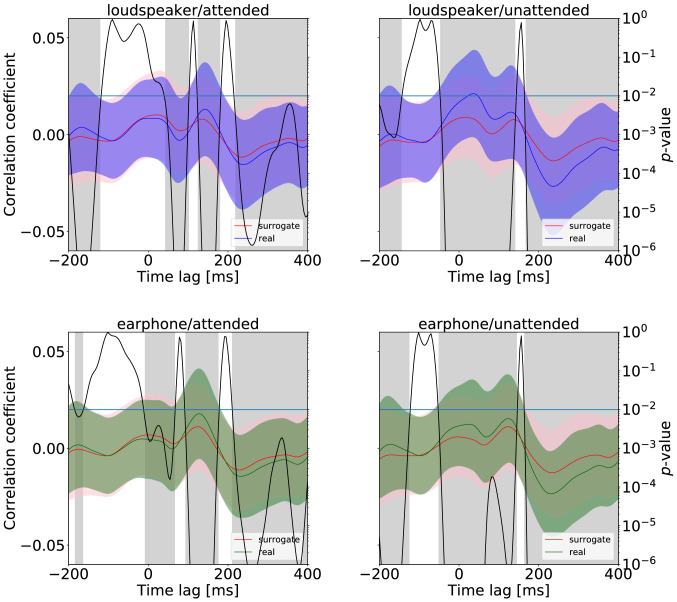
Results of the *t* test between the surrogate distribution and the real cross-correlation function for all conditions. The cross-correlation functions shown as green and blue curves are averaged real distributions, and the ones shown as red curves are the average of the surrogate distributions. Black curves represent the *p* value. The gray-filled area indicates $p < 0.01$

We show the results of the *t* test between the cross-correlation function and surrogate distribution for four conditions in Fig. 7. In Fig. 7, we can observe that three peaks (the first and second positive peaks followed by the negative peak) are mostly in the positive time interval, with $p<0.01$ (indicated with the gray shadow). The first positive peaks are found in a statistically significant interval between $-46.9$ and 140.6 ms for the loudspeaker and unattended condition, between $-7.8$ and 66.4 ms for the earphone and attended condition, and between $-50.8$ and 144.5 ms for the earphone and unattended condition. However, for the loudspeakers and attended condition, the first peak is not in the statistically significant interval. The second positive peaks and following negative peaks are found in a statistically significant interval for all conditions; the second positive peaks are between 125.0 and 179.7 ms for the loudspeaker and attended condition, between 168.0 and 441.4 ms for the loudspeaker and unattended condition, between 93.8 and 175.8 ms for the earphone and attended condition, and between 164.1 and 425.8 ms for the earphone and unattended condition. The negative peaks are in a statistically significant interval between 218.8 and 425.8 ms for the loudspeaker and attended condition, between 168.0 and 441.4 ms for the loudspeaker and unattended condition, between 210.9 and 418.0 ms for the earphone and attended condition, and between 164.1 and 425.8 ms for the earphone and unattended condition.

**Fig. 8:**
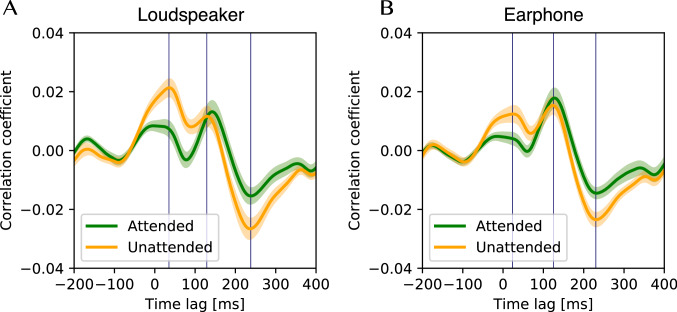
The cross-correlation function averaged across participants. The graph shows the cross-correlation function averaged across participants, trials, and electrodes. The filled area represents the standard deviation among the participants. Correlation with the melody that was paid attention to is shown in green, and with the melody that was not paid attention to in orange

Next, the cross-correlation functions averaged across all electrodes, all trials, and all participants are shown in Fig. 8. After the onset ($\tau =0$), we observed two positive peaks at $\tau =35.16$ and $\tau =128.9$ (the loudspeaker presentation) and $\tau =23.44$ and $\tau =125.0$ (the earphone presentation), respectively, and negative peaks at $\tau =238.3$ (the loudspeaker presentation) and $\tau =230.5$ (the earphone presentation). The first positive and the negative peak indicate that unattended melodies are more correlated than attended ones in terms of the mean values. A two-way repeated-measure ANOVA validated these observations to analyze the effect of presentation methods (loudspeaker and earphone) and attention (attended and unattended) on the magnitude of the first positive, the second positive, and the negative peaks.

For the first positive peak, simple main effects analysis showed the effect of attention (attended and unattended) was statistically significant ($F(1, 13)=44.500$; $p<0.001$; $\eta ^2=0.501$) and the effect of presentation methods (loudspeaker and earphone) was also significant ($F(1, 13)=23.071$; $p<0.001$; $\eta ^2=0.160$). Although both effects of attention and presentation are significant, the effect size $\eta ^2$ for presentation methods is much smaller than that for attention. On the other hand, there was not a statistically significant interaction between the effects of presentation and attention ($F(1, 13) = 4.246$; $p = 0.060$; $\eta ^2=0.026$).

For the second positive peak, only the effect of presentation methods (loudspeaker and earphone) showed significant differences ($F(1, 13)=12.623$; $p=0.004$; $\eta ^2=0.142$). There was no significant difference in the effect of attention ($F(1, 13)=-0.150$; $p=0.704$; $\eta ^2=0.008$), and there was no significant interaction ($F(1, 13) = 4.109$; $p = 0.064$; $\eta ^2=0.013$).

For the negative peak, the result showed a significant difference in the effect of attention ($F(1, 13)=38.613$; $p<0.001$; $\eta ^2=0.495$), but there was no significant difference ($F(1, 13)=1.383$; $p=0.261$; $\eta ^2=0.027$) and no significant interaction ($F(1, 13) = 2.138$; $p = 0.167$; $\eta ^2=0.009$).

**Fig. 9:**
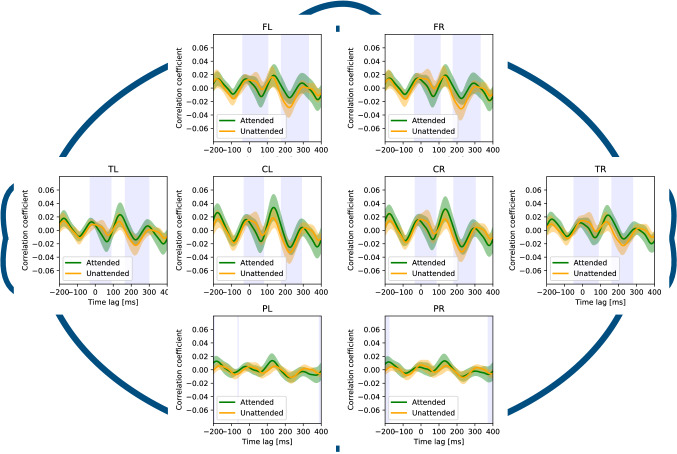
Results of *t* test of each region and the cross-correlation functions. We highlighted peak times showing the significant difference ($p<0.01$, *t* test). FR represents the right side of the frontal area, and FL represents the left side of the frontal area. Similarly, CR is the right central, CL is the left central, TR is the right temporal, TL is the left temporal, PR is the right parietal and occipital, and PL is the left parietal and occipital

Finally, results of the *t* test at eight regions (frontal left/right, central left/right, temporal left/right, and parietal left/right) are illustrated in Fig. 9. We highlighted peak times showing the significant difference ($p<0.01$,* t* test) in the figures due to attention in the frontal, central, and temporal regions in the latency corresponding to the first positive and the negative peaks, respectively. There were no significant differences in the parietal and occipital areas.

### Classification based on common spatial patterns

**Fig. 10:**
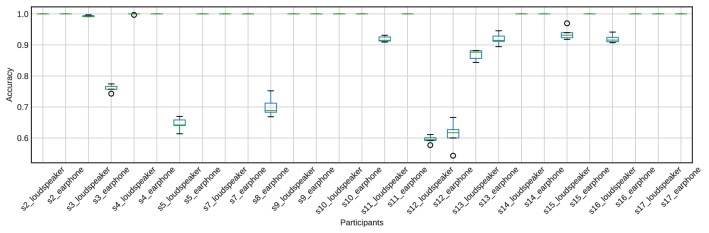
The accuracy of attention detection by SVM using the EEG signal. This is the result of fivefold cross-validation. We extracted the features from the EEG signals for 1.25 s by CSP

**Fig. 11:**
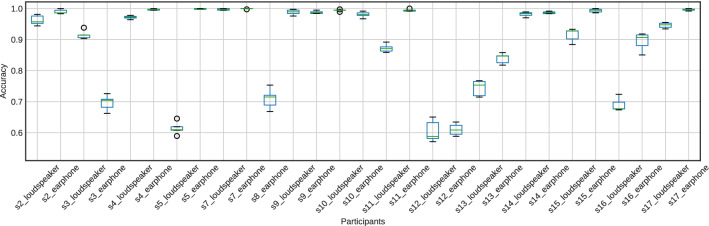
The accuracy of attention detection by SVM using the cross-correlation function. This is the result of fivefold cross-validation. We extracted the features from the cross-correlation function by CSP

Figures 10 and 11 show the accuracy of fivefold cross-validation using SVM with the features extracted using the CSP method. The accuracy was much higher than the chance level (50%) for all participants. Furthermore, CSP features from EEG signals achieved 100% accuracy for 11 of the 15 participants (s2, s4, s5, s7, s9, s10, s11, s14, s15, s16, and s17). Also, CSP features from the cross-correlation function achieved 98% accuracy for 10 of the 15 participants (s2, s4, s5, s7, s9, s10, s11, s14, s15, and s17). The average accuracy across participants when using EEG signals was 0.926, and the average accuracy using the cross-correlation function was 0.892. Using EEG signals tends to have higher accuracy than using the cross-correlation function, as shown in Figs. 10 and 11.

**Fig. 12:**
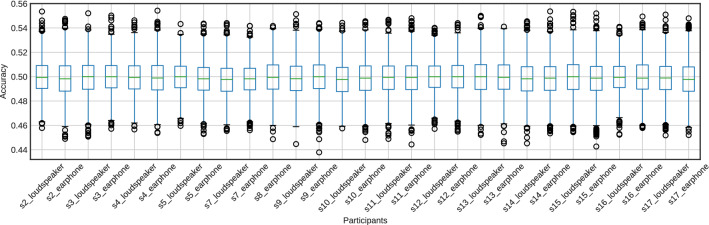
Box plots of the classification accuracy for the randomly shuffled datasets of the features extracted by the CSP from the EEG signals for 1.25 s

**Fig. 13:**
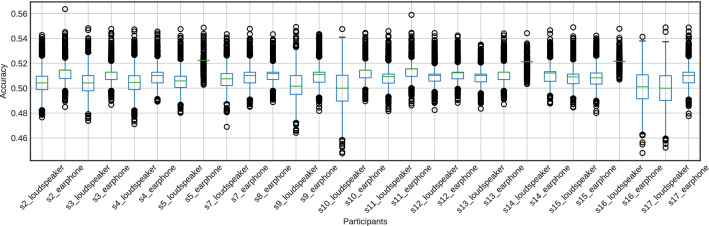
Box plots of the classification accuracy for the randomly shuffled datasets of the features extracted by the CSP from the cross-correlation function

The accuracy of 2000 repetitions of fivefold cross-validation using randomly labeled data is shown in Figs. 12 and 13. The accuracy was around the chance level (50%) for all participants.

## Discussion

We conducted the experiment to test two hypotheses on the effect of selective attention to music in EEG signals when a human listens to two musical excerpts of different tempos simultaneously. The experiment did not support the first hypothesis that the cross-correlation of EEG signals with the attended music is stronger than that with the unattended music; we even found the opposite. In a latency of about 0–100 ms and 200–300 ms, unattended melodies were more correlated than attended. The results suggest that the positive peak ($\sim 50$ ms) reflects the suppression of the processing on timbre, and the negative peak ($\sim 240$ ms) reflects the suppression of the processing on melodies and rhythm. The above observation contrasts with speech results (Horton et al. 2013; Kong et al. 2014).

The second hypothesis was that the spatial modulation of the response is different depending on attentional conditions under multiple music presentations. The results show that this is the case. Furthermore, CSP features seen in EEG signals achieved 100% accuracy for 11 of the 15 participants. Also, CSP features from the cross-correlation function achieved 98% accuracy for 10 of the 15 participants. Our findings suggest using the spatial distribution of EEG signals is sufficient to predict attended music and speech.

### Influence of selective attention on neural entrainment

As shown in Fig. 6, frequency response peaks were found at harmonics of the tempo, indicating that neural entrainment occurred while listening to melodies, which has been observed in previous studies (Meltzer et al. 2015; Kaneshiro et al. 2020). Also, we can be sure that the result was not chance because there was a significant difference between the surrogate distribution and real cross-correlation function, as shown in Fig. 7. In particular, a first positive peak was at $\sim 50$ ms, a second positive peak was at $\sim 150$ ms, and a negative peak was at $\sim 240$ ms.

**Fig. 14:**
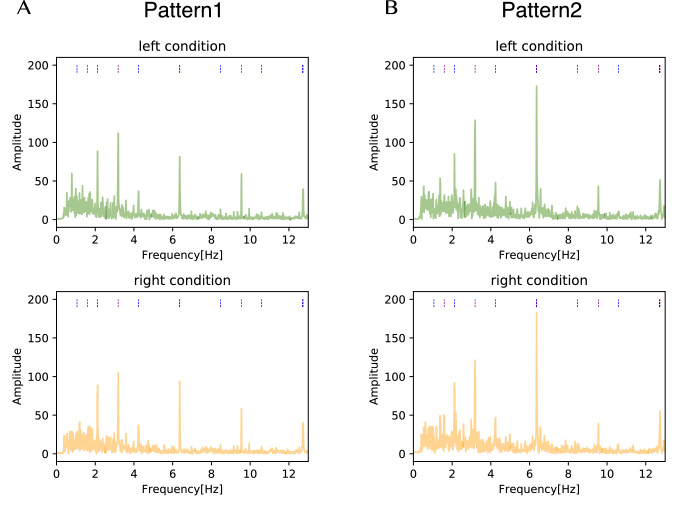
Spectra of the envelope of the auditory stimulus when considering the time shift of the melody. Green shows the spectra of the envelope of the auditory stimulus in the left attention condition and orange in the right attention condition. Auxiliary lines are drawn vertically at harmonic frequencies to the melody’s tempo under the top of each figure. The auxiliary line for the harmonics frequencies of the melody tempo presented from the left side is blue. The auxiliary line for the harmonics frequencies of the melody tempo presented from the right side is purple

As for the frequency responses related to beat in the stimuli, Fig. 6 demonstrates that dominant peaks occurred at 6.4 Hz, even though the fundamental tempos of the melodies were at 2.1 and 3.2 Hz. The frequency of 6.4 Hz was the third harmonic of the tempo of the left melody and the second harmonic of the tempo of the right melody. The above observations can be interpreted by looking at the characteristics of the stimuli. As shown in Fig. 14, the peak value at 6.4 Hz in the envelope spectrum of the musical stimulus was more prominent than that at the fundamental tempo in Pattern 2. Also, in Pattern 1, the peak value at 6.4 Hz was similar to that at the fundamental tempo. In particular, the prominent peak in Pattern 2 may be caused by the right melody of Pattern 2 from “Csikos Post,” with many eighth notes corresponding to 6.4 Hz. Thus, it is reasonable to interpret that the characteristics at 6.4 Hz in the frequency domain of both patterns were directly reflected in the EEG responses.

As illustrated in Fig. 8, the results of the cross-correlation functions showed a significant difference between the attended and unattended conditions depending on the time latency. Regarding the first positive peak ($\sim 50$ ms) and negative peak ($\sim 240$ ms), the peak value in the unattended condition was larger than in the attended condition. Contrary to our results, it has often been reported that attention causes larger EEG responses in the speech and beat domain (Hill et al. 2005; Horton et al. 2013; Kong et al. 2014; Sato et al. 2019), indicating that our result is the opposite of these previous results. This may imply that the music perception process related to selective attention would differ from speeches or beats. A recent imaging study reported that asymmetry between speech and music was found in the effective area for the listening sound classification task from the neural response (Albouy et al. 2020). Our result seems to show that attention in the music domain may cause a suppression of the perception steps. Lakatos et al. (2013) reported that excitability increases when the stimulus frequency matches the best frequency (BF) of a site in the primary auditory cortex (A1), whereas excitability decreases at nonmatching sites (counter-phase entrainment). Thus, the difference in the excitability phase to non-BF and BF in A1 sites might have affected the observed EEG entrainment when music, which is more frequency-complex than speech or beat sounds, was presented. In any case, it is difficult to explain why the present experiment obtained the observation of weakened entrainment.

The spatial distribution of the cross-correlation function illustrated in Fig. 9 indicates that this suppression occurred in the frontal, central, and temporal areas. Regarding the music perception process, as Koelsch (2011) reported, auditory features such as timbre are extracted at a latency of 10–100 ms, and then melodies and rhythm are processed, with each peak reflecting each process. Applying the above finding to our results, the positive peak ($\sim 50$ ms) would indicate the suppression of the processing on timbre, and the negative peak ($\sim 240$ ms) would indicate the suppression of the processing on melodies and rhythm. Depending on the processing latency, selective attention may affect EEG response differently.

### EEG modulation while listening to two different melodies

We compared the spectral density of the EEG signals (Fig. 6) with the spectrum of the envelope of the stimuli (Fig. 14). In general, the peaks of the spectral density of the EEG signals matched that of the presented stimuli (Meltzer et al. 2015; Kaneshiro et al. 2020). However, the EEG spectral showed peaks at frequencies of about 5.3 Hz and about 7.4 Hz, which was not observed in the original Pattern 1. These peaks occurred not only in the loudspeakers presentation but also in the earphone presentation. Therefore, they were not caused by nonlinear distortions in the ear, such as an otoacoustic emission (Kemp 1978), but rather by cross-modulation during the processing of the two presented stimuli in the brain. Several reports have observed cross-modulation in EEG signals when multiple stimuli were presented simultaneously (Nozaradan et al. 2011; Matsumoto and Tanaka 2018; Vergeer et al. 2018). Matsumoto and Tanaka (2018) reported that when they presented auditory (frequency $f_1$) and visual (frequency $f_2$) stimuli simultaneously, the cross-modulation of $f_1+f_2$ appeared in the EEG signals. Our results suggest that cross-modulation occurs when two auditory stimuli are presented. Also, it is unclear whether the attention induced cross-modulation. This topic is worth investigating in the future.

### Attention detection based on spatial modulation in the alpha band of EEG signals

To our knowledge, the present paper is the first report to detect attended music consisting of several musical notes only with EEG signals. Previous studies reporting classifications of selective attention to musical excerpts have used modified music consisting of single notes (Meltzer et al. 2015) or amplitude-modulated music enhancing the beats (Sato et al. 2019). Our approach achieved 98% accuracy for 10 of the 15 participants using features from the cross-correlation function between the music excerpts and measured EEG signals. Moreover, features extracted from only EEG signals with a length of 1.25 s achieved 100% accuracy for 11 of the 15 participants. In the speech domain, Cai et al. (2021) recently reported using CSP-based features from a 1-s EEG signal in attention detection together with convolutional neural networks; this resulted in an accuracy of 82.8%. Therefore, using a more sophisticated classifier, including deep neural networks, might enhance our classification analysis.

The current paper exhibited that attention detection to music was achieved based on the spatial modulation of alpha-band EEG signals alone, suggesting that attentional control may cause the spatial modulation of alpha rhythms. Also, using alpha-band EEG signals tends to have higher accuracy than using the cross-correlation function between the stimuli and EEG signals. Our finding suggests that using only EEG signals might be effective in attention detection for music and other modalities such as speech, even though recent reports on attention detection of speech (Cai et al. 2021; Geravanchizadeh and Roushan 2021) have used the stimuli (speech) and EEG signals simultaneously.

Regarding selective attention in the auditory, Kerlin et al. (2010) suggested that attention to speech modulates the alpha band of EEG signals. It has also been shown that this modulation is associated with selecting attentional direction (Deng et al. 2020). CSP analysis has suggested that alpha band modulation also occurs for selective attention to music played simultaneously. Regarding selective attention, studies on visual attention (Hong et al. 2015) and visual-auditory attention (Kumagai et al. 2018) have also pointed out that it causes modulation of the alpha band. The present study’s and previous studies’ results support the finding that neural synchronization of the alpha band in the cerebrum serves as a “switch” for selecting attention (Foxe and Snyder 2011). On the other hand, it should be noted that it is hard to conclude a causal relationship between selective attention and alpha band modulation. A recent study has pointed out that alpha power does not bring about selective attention concerning visual attention (Antonov et al. 2020).

### Effects of experimental design

The following experimental design parameters may have influenced the results. First, the following two parameters are the left-right differences in the presented stimuli: musical instruments and tempo. It has been suggested that brain activity and behavioral results during music listening may be affected depending on musical instruments and tempo (Treder et al. 2014; Soontreekulpong et al. 2018). The behavioral results in Fig. 5 suggest some disadvantages to the right-attended condition. This result may not be due to the left-right difference in room acoustics since we used a well-designed soundproof room for music practice. This result may be dependent on the choice of the right melody. It is a future task to investigate brain activity during selective attention when listening to music for each parameter.

In addition, three other parameters may also affect the results: presentation methods, familiarity with the musical excerpts, and musical experience. As for the presentation methods, we used loudspeakers on the first day and earphones on the second day. We considered the possibility that the presentation method of the stimuli may have affected selective attention, but this is not the topic of the current paper. Also, familiarity could have been a factor that affected the results of attention detection; indeed, Kumagai et al. (2018) reported that familiarity with musical excerpts affected the strength of neural entrainment. Finally, in the current study, musical amateurs participated, and the level of musical experience was not considered a factor. It has been reported that participants who have much more musical experience are better at auditory attention tasks using beats than ones with little experience of music (Laffere et al. 2020; Putkinen et al. 2021). Future research is expected on the effects of musical experience and presentation methods on selective attention in musical listening.

In our experimental setup, different instruments were presented at different tempos to facilitate the separation of the two music streams. In addition, we started presenting the attended stream one second earlier than the unattended stream so that the attention could be continuously directed to the attentional stream. The result showed that the silence intervals were detected reasonably accurately. However, our study did not examine whether the two music streams could be separated and did not address the mechanism that allows attention to be directed to one of the two music streams. For example, familiarity with music may affect the result of the silent interval detection; we guess that the detection rate of silent intervals may decrease when unfamiliar music is used. Further studies are necessary.

## Conclusion

The present paper investigated the effects of selective attention on neural entrainment and alpha band spatial modulation under multiple music presentations. We measured EEG signals during selective attention to one of two melodies. Our observations on the cross-correlation function between the envelope of the melody and EEG signals show attention to the melody as inducing suppression of neural entrainment under multiple music presentations, which is contrary to the speech domain (Horton et al. 2013). In order to further improve the reliability of these results, it would seem necessary to test them in more depth with more superficial musical stimuli. Also, it would be interesting to use music consisting of melodies with vocals to see the difference in response to melody and speech. Moreover, we found that machine learning using spatial modulation of the alpha band EEG signals efficiently identifies the attended melody. This suggests that the spatial modulation of the alpha band occurs in conjunction with selective attention. It remains unclear whether this alpha modulation was caused by simple attention to music or directional attention to the location of the sound. A mixed music signal played with a single loudspeaker would be a possible solution to the problem, but this is an open problem for future work.

## Data Availability

The datasets generated for this study can be found in online repositories(https://github.com/ttlabtuat/musicattentioneeg).

## Footnotes

1 The moment at which the first derivative of the analog input signal of the beep exceeds a particular threshold value was set to “onset.”

2 The correlation was measured using a Pearson coefficient with a threshold of 0.5. The average number of discarded components was 2.43 for all participants.

## References

[CR1] Albouy P, Benjamin L, Morillon B, Zatorre RJ (2020). Distinct sensitivity to spectrotemporal modulation supports brain asymmetry for speech and melody. Science.

[CR2] Alho K, Medvedev SV, Pakhomov SV, Roudas MS, Tervaniemi M, Reinikainen K, Zeffiro T, Näätänen R (1999). Selective tuning of the left and right auditory cortices during spatially directed attention. Cogn Brain Res.

[CR3] Antonov PA, Chakravarthi R, Andersen SK (2020). Too little, too late, and in the wrong place: alpha band activity does not reflect an active mechanism of selective attention. NeuroImage.

[CR4] Baltzell LS, Srinivasan R, Richards V (2019). Hierarchical organization of melodic sequences is encoded by cortical entrainment. NeuroImage.

[CR5] Belouchrani A, Abed-Meraim K, Cardoso J, Moulines E (1993) Second-order blind separation of temporally correlated sources. In: Proceedings of international conference of digital signal processing, pp 346–351

[CR6] Bregman AS (1994). Auditory scene analysis: the perceptual organization of sound.

[CR7] Cai S, Li P, Su E, Xie L (2021). Auditory attention detection via cross-modal attention. Front Neurosci.

[CR8] Choi I, Rajaram S, Varghese LA, Shinn-Cunningham BG (2013). Quantifying attentional modulation of auditory-evoked cortical responses from single-trial electroencephalography. Front Hum Neurosci.

[CR9] Deng Y, Choi I, Shinn-Cunningham B (2020). Topographic specificity of alpha power during auditory spatial attention. NeuroImage.

[CR10] Dietrich A, Kanso R (2010). A review of EEG, ERP, and neuroimaging studies of creativity and insight. Psychol Bull.

[CR11] Doelling KB, Poeppel D (2015). Cortical entrainment to music and its modulation by expertise. Proc Natl Acad Sci.

[CR12] Dong Y, Raif KE, Determan SC, Gai Y (2017). Decoding spatial attention with EEG and virtual acoustic space. Physiol Rep.

[CR13] Foxe JJ, Snyder AC (2011). The role of alpha-band brain oscillations as a sensory suppression mechanism during selective attention. Front Psychol.

[CR14] Geravanchizadeh M, Roushan H (2021). Dynamic selective auditory attention detection using RNN and reinforcement learning. Sci Rep.

[CR15] Hill NJ, Lal TN, Bierig K, Birbaumer N, Schölkopf B (2005) An auditory paradigm for brain-computer interfaces. Adv Neural Inf Process Syst pp. 569–576

[CR16] Hong X, Sun J, Bengson JJ, Mangun GR, Tong S (2015). Normal aging selectively diminishes alpha lateralization in visual spatial attention. NeuroImage.

[CR17] Horton C, D’Zmura M, Srinivasan R (2013). Suppression of competing speech through entrainment of cortical oscillations. J Neurophysiol.

[CR18] Horton C, Srinivasan R, D’Zmura M (2014). Envelope responses in single-trial EEG indicate attended speaker in a ‘cocktail party’. J Neural Eng.

[CR19] Kaneshiro B, Nguyen DT, Norcia AM, Dmochowski JP, Berger J (2020). Natural music evokes correlated EEG responses reflecting temporal structure and beat. NeuroImage.

[CR20] Karns CM, Isbell E, Giuliano RJ, Neville HJ (2015). Auditory attention in childhood and adolescence: an event-related potential study of spatial selective attention to one of two simultaneous stories. Dev Cogn Neurosci.

[CR21] Kemp DT (1978). Stimulated acoustic emissions from within the human auditory system. J Acoust Soc Am.

[CR22] Kerlin JR, Shahin AJ, Miller LM (2010). Attentional gain control of ongoing cortical speech representations in a cocktail part. J Neurosci.

[CR23] Koelsch S (2011). Toward a neural basis of music perception-a review and updated model. Front Psychol.

[CR24] Kong YY, Mullangi A, Ding N (2014). Differential modulation of auditory responses to attended and unattended speech in different listening conditions. Hear Res.

[CR25] Kumagai Y, Matsui R, Tanaka T (2018). Music familiarity affects EEG entrainment when little attention is paid. Front Hum Neurosci.

[CR26] Laffere A, Dick F, Tierney A (2020). Effects of auditory selective attention on neural phase: Individual differences and short-term training. NeuroImage.

[CR27] Lakatos P, Musacchia G, O’Connel MN, Falchier AY, Javitt DC, Schroeder CE (2013). The spectrotemporal filter mechanism of auditory selective attention. Neuron.

[CR28] Lalor EC, Power AJ, Reilly RB, Foxe JJ (2022). Resolving precise temporal processing properties of the auditory system using continuous stimuli. J Neurophysiol.

[CR29] Liu Y, Bengson J, Huang H, Mangun GR, Ding M (2016). Top-down modulation of neural activity in anticipatory visual attention: control mechanisms revealed by simultaneous EEG-fMRI. Cereb Cortex.

[CR30] Matsui R, Tanaka T (2019) Effect on entrainment by selective attention to music and speech. In: Society for neuroscience (SfN), Chicago

[CR31] Matsumoto A, Tanaka T (2018) Cross-modulation response underlying sensorimotor synchronization to auditory and visual beats. In: 2018 IEEE international conference on systems, man, and cybernetics (SMC), IEEE, pp 609–614

[CR32] Meltzer B, Reichenbach CS, Braiman C, Schiff ND, Hudspeth A, Reichenbach T (2015). The steady-state response of the cerebral cortex to the beat of music reflects both the comprehension of music and attention. Front Hum Neurosci.

[CR33] Nozaradan S, Peretz I, Missal M, Mouraux A (2011). Tagging the neuronal entrainment to beat and meter. J Neurosci.

[CR34] Pugh KR, Shaywitz BA, Shaywitz SE, Fulbright RK, Byrd D, Skudlarski P, Shankweiler DP, Katz L, Constable RT, Fletcher J (1996). Auditory selective attention: an fmri investigation. NeuroImage.

[CR35] Putkinen V, Saarikivi K, Chan TMV, Tervaniemi M (2021). Faster maturation of selective attention in musically trained children and adolescents: Converging behavioral and event-related potential evidence. Eur J Neurosci.

[CR36] Ramoser H, Muller-Gerking J, Pfurtscheller G (2000). Optimal spatial filtering of single trial EEG during imagined hand movement. IEEE Trans Rehab Eng.

[CR37] Sato TG, Shiraki Y, Moriya T (2019) Detecting attention shift from neural response based on beat-frequency-modulated musical excerpts. In: ICASSP 2019-2019 IEEE international conference on acoustics, speech and signal processing (ICASSP), pp. 1110–1114

[CR38] Soontreekulpong N, Jirakittayakorn N, Wongsawat Y (2018) Investigation of various manipulated music tempo for reducing negative emotion using beta EEG index. In: 2018 international electrical engineering congress (IEECON), IEEE, pp. 1–4

[CR39] Stupacher J, Wood G, Witte M (2017). Neural entrainment to polyrhythms: a comparison of musicians and non-musicians. Front Neurosci.

[CR40] Sturm I, Blankertz B, Potes C, Schalk G, Curio G (2014). ECoG high gamma activity reveals distinct cortical representations of lyrics passages, harmonic and timbre-related changes in a rock song. Front Hum Neurosci.

[CR41] Treder MS, Purwins H, Miklody D, Sturm I, Blankertz B (2014). Decoding auditory attention to instruments in polyphonic music using single-trial EEG classification. J Neural Eng.

[CR42] Trost W, Frühholz S, Schön D, Labbé C, Pichon S, Grandjean D, Vuilleumier P (2014). Getting the beat: entrainment of brain activity by musical rhythm and pleasantness. NeuroImage.

[CR43] Vergeer M, Kogo N, Nikolaev AR, Alp N, Loozen V, Schraepen B, Wagemans J (2018). EEG frequency tagging reveals higher order intermodulation components as neural markers of learned holistic shape representations. Vis Res.

[CR44] Wollman I, Arias P, Aucouturier JJ, Morillon B (2020). Neural entrainment to music is sensitive to melodic spectral complexity. J Neurophysiol.

[CR45] Zoefel B, VanRullen R (2016). EEG oscillations entrain their phase to high-level features of speech sound. NeuroImage.

